# CUDC-907 inhibits glioblastoma and enhances glioblastoma sensitivity to temozolomide by inhibiting DNA damage repair

**DOI:** 10.1016/j.gendis.2025.101948

**Published:** 2025-11-24

**Authors:** Chencheng Fang, Pan Gou, Dandan Zhang, Xuanxuan Wu, Xiao Li, Man Li, Lu Gan, Jinjin Luo, Hongjuan Cui, Man Xu, Ping Liang

**Affiliations:** a Department of Neurosurgery, Children’s Hospital of Chongqing Medical University, National Clinical Research Center for Child Health and Disorders, Ministry of Education Key Laboratory of Child Development and Disorders, Chongqing Key Laboratory of Child Neurodevelopment and Cognitive Disorders, Chongqing 400014, China; b Jinfeng Laboratory, Chongqing 401329, China; c Medical Research Institute, State Key Laboratory of Resource Insects, Southwest University, Chongqing 400715, China

**Keywords:** Chemosensitivity, CUDC-907, Glioblastoma, Organoid, Temozolomide

## Abstract

CUDC-907, referred to as Fimepinostat, functions as a dual inhibitor of PI3K and HDAC, exhibiting significant anti-tumor efficacy in a range of cancer types. However, its specific role in glioblastoma is not well understood. In this study, we investigated the effects of CUDC-907 on glioblastoma using cellular, organoid, and animal models to assess its inhibitory potential and toxicity. *In vitro*, we assessed glioblastoma cell proliferation, migration, invasion, and apoptosis using standard assays. glioblastoma organoids were treated to examine three-dimensional tumor growth and cellular changes. For *in vivo* analysis, animal models with glioblastoma received CUDC-907 to study its systemic impact and toxicity, with tumor progression closely monitored. We also tested the synergy between CUDC-907 and temozolomide to evaluate enhanced chemosensitivity. RNA sequencing was conducted to explore the fundamental molecular mechanisms involved, focusing on alterations in the cell cycle and DNA damage repair pathways. Our findings show that CUDC-907 significantly suppresses the proliferation, migration, and invasion of glioblastoma cells and promotes apoptosis, while exhibiting minimal toxicity. Additionally, CUDC-907 acts synergistically with temozolomide, a chemotherapy drug used for glioblastoma treatment, enhancing glioblastoma’s chemosensitivity to temozolomide. RNA sequencing suggests that CUDC-907 achieves its effects by influencing the glioblastoma cell cycle and inhibiting DNA damage repair. Overall, the data suggest that CUDC-907 may be a promising anti-cancer agent for glioblastoma treatment.

## Introduction

Glioblastoma (GBM) is widely acknowledged as the most prevalent form of malignant primary brain tumor, accounting for approximately 50.9% of all primary malignant tumors within the central nervous system.^1^ The conventional treatment protocol for patients recently diagnosed with GBM generally includes surgical removal of the tumor, followed by a regimen of radiotherapy and chemotherapy.^2^ However, it is crucial to note that not all patients demonstrate a favorable response to these conventional therapeutic strategies. Despite extensive efforts aimed at enhancing clinical outcomes through combination therapies that integrate surgery, radiotherapy, systemic treatments (including chemotherapy and targeted therapies), and supportive care, the overall survival rates remain unacceptably low. Studies indicate that the median survival duration for patients with GBM ranges from 15 to 18 months,^3^, ^4^, ^5^ with a relative survival rate of merely 6.9% at five years post-diagnosis.^6^ Consequently, the treatment options available for individuals with GBM are severely limited, underscoring an urgent need for the development of novel and innovative therapeutic strategies specifically tailored to address the challenges posed by this aggressive malignancy.

The phosphoinositide 3-kinase (PI3K) signaling pathway is pivotal in oncological studies, with its dysregulation being significantly linked to tumor development, advancement, and resistance to therapeutic interventions. This signaling pathway facilitates the transformation of phosphatidylinositol 4,5-bisphosphate (PIP2) into phosphatidylinositol 3,4,5-trisphosphate (PIP3) through the action of PI3K, subsequently activating downstream effectors, protein kinase B (AKT) and mammalian target of rapamycin (mTOR), which govern essential biological functions, including cell proliferation, metabolic processes, and cell survival. Moreover, the tumor suppressor known as phosphatase and tensin homolog (PTEN) acts as a vital negative regulator of the PI3K signaling pathway, thereby preserving the equilibrium of cellular signaling in relation to tumor development.^7^^,^^8^ Recent genomic studies in human cancer have identified frequent targeting of PI3K pathway components by germline or somatic mutations across various cancers. These insights, coupled with the susceptibility of PI3K and related kinases to pharmacological intervention, make this pathway a compelling target for cancer therapy.^9^ However, the effectiveness of PI3K inhibitors is constrained by the concurrent activation of alternative survival and growth pathways.^10^^,^^11^

**Figure S1:**
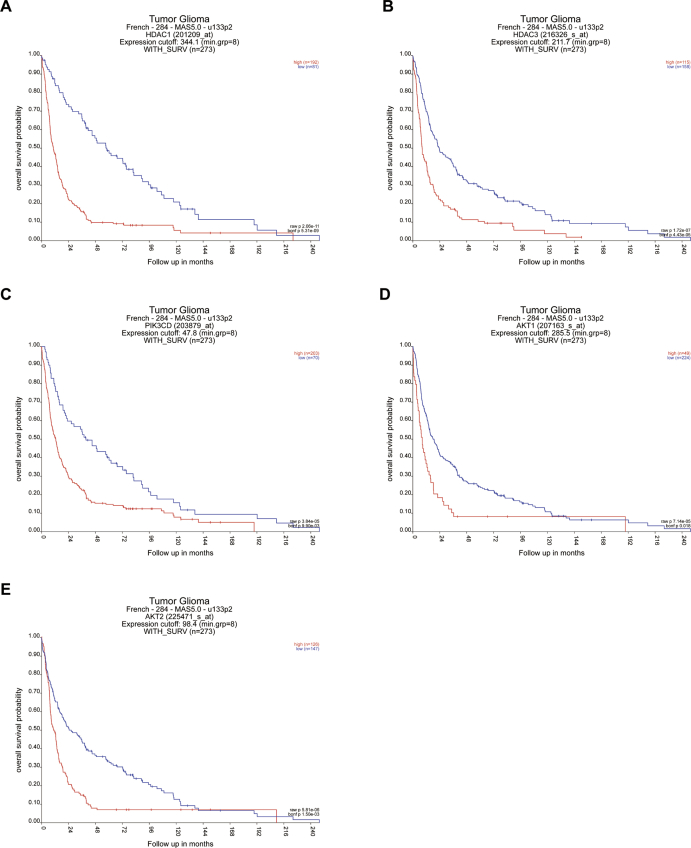
High expression levels of PI3K and HDAC-related genes are correlated with poor patient prognosis. **(A**–**E)** Kaplan–Meier analysis of progression-free survival was conducted using data from the Tumor Glioma French 284 database, with *P*-values obtained from the log-rank test indicated.

A viable approach to address the inherent limitations of directly targeting the PI3K pathway involves the inhibition of histone deacetylases (HDACs) to disrupt multiple signaling pathways simultaneously.^12^ By influencing both histone and non-histone substrates, HDAC inhibitors can impact a range of cellular functions and may enhance the therapeutic effects of PI3K inhibitors through synergistic interactions.^13^ Elevated expression of PI3K and HDAC-related genes is significantly negatively correlated with the prognosis of GBM patients (Fig. S1A–S1E). Additionally, mutations in PI3K-related genes are associated with reduced overall survival.^14^, ^15^, ^16^

CUDC-907 was first reported in 2012 by the research team led by Rudi Bao. This compound integrates the active hydroxamic acid functional group characteristic of HDAC inhibitors with a PI3K inhibitory component within a unified molecular framework, establishing it as a novel dual inhibitor targeting both PI3K and HDAC.^17^ As a standalone small molecule, CUDC-907 exhibits considerable promise in inhibiting cancer proliferation and metastasis through the concurrent disruption of various oncogenic signaling pathways. Current research has validated the effectiveness of CUDC-907 across a range of cancers, such as pancreatic cancer, prostate cancer, B-cell lymphoma, thyroid cancer, Burkitt lymphoma, and neuroblastoma.^18^, ^19^, ^20^, ^21^, ^22^, ^23^

Since its FDA approval in 2005, temozolomide (TMZ) has become the primary chemotherapeutic agent for patients diagnosed with GBM.^24^ In a pivotal study, Stupp et al demonstrated that the combination of TMZ with radiation therapy resulted in a median overall survival extension of two months, representing a significant advancement in the treatment of GBM with this novel chemotherapeutic agent. Consequently, TMZ has become a cornerstone of GBM therapy. However, it is essential to recognize that TMZ is associated with tumor resistance and recurrence. The extensive use of TMZ, combined with the highly heterogeneous and mutation-prone characteristics of GBM, frequently leads to the development of resistance in these aggressive tumors. TMZ methylates the N7 and O6 positions of guanine and the N3 position of adenine in DNA or RNA.^25^ These methylated sites can persist as mutations, be repaired by the mismatch repair (MMR) system, be removed by base excision repair (BER) enzymes like alkylpurine-DNA-N-glycosylase (APNG), or be demethylated by O6-methylguanine methyltransferase (MGMT). Active MMR increases TMZ sensitivity, while resistance is linked to MGMT, APNG, and BER proteins. Worryingly, over 50% of GBM patients undergoing treatment with TMZ do not demonstrate a positive therapeutic response.^26^ This issue of TMZ resistance presents a substantial challenge that must be addressed to improve treatment outcomes for individuals affected by GBM.^27^

Therefore, we hypothesize that CUDC-907 alone can inhibit GBM and synergize with TMZ to enhance its sensitivity. In this study, we describe how CUDC-907 can inhibit the growth, proliferation, migration, and invasion of GBM cells, induce cell apoptosis, and induce cell cycle arrest by down-regulating MYC. Additionally, we demonstrate that CUDC-907 and TMZ can synergistically inhibit the malignant behavior of GBM cells. CUDC-907 reduces GBM resistance to TMZ by inhibiting DNA damage repair mechanisms activated by TMZ.

## Materials and methods

### Cell culture

Cell lines, including LN-229 (RRID:CVCL_0393), A172 (RRID:CVCL_0131), T98G (RRID:CVCL_0556), and SVG p12 (RRID:CVCL_3797), were sourced from ATCC. A primary GBM cell line, referred to as GBM1, was created from tumor samples collected from a patient diagnosed with GBM. All of the GBM cell lines, along with the primary GBM line, were maintained in Dulbecco’s modified Eagle’s medium (DMEM) (Gibco, New York, USA). The culture medium was enriched with 10% fetal bovine serum and 1% penicillin-streptomycin. All cell lines were cultured at 37 °C in a humidified atmosphere of 5% CO_2_. We certify that all human cell lines have been authenticated using STR profiling within the last three years.

### Antibodies and reagents

Akt (#9272), phospho-Akt (Ser473) (#4060), and phospho-histone H2A.X (Ser139) (#9718) antibodies were purchased from Cell Signaling Technology (Massachusetts, USA). c-MYC (10828-1-AP), HDAC1 (10197-1-AP), HDAC2 (12922-3-AP), HDAC3 (81211-1-RR), H3 (17168-1-AP), acetyl-histone H3 (Lys27) (82902-1-RR), GAPDH (10494-1-AP), CDK2 (10122-1-AP), Tubulin (14555-1-AP), P21 (10355-1-AP), PARP1 (13371-1-AP), Vimentin (60330-1-IG), MMP2 (66366-1-IG), N-cadherin (22018-1-AP), and Caspase 3 (82202-1-RR) antibodies were obtained from Proteintech Group (Wuhan, China). SOX2 (ab79351) antibody was purchased from Abcam (Massachusetts, USA). CDK4 (sc-53638) antibody was purchased from Santa Cruz (California, USA). Ki67 (MA5-14520) antibody was purchased from Invitrogen (California, USA). Cell Cycle kit, Apoptosis kit, and EdU kit were acquired from Beyotime (Shanghai, China). CUDC-907 (S2759) was purchased from Selleck (Texas, USA). TMZ (M2129) was purchased from Abmole (Shanghai, China).

### Cell proliferation analysis

Using the CCK8 experiment and Incucyte SX5 (Sartorius, Niedersachsen, Germany), cell viability was evaluated. Cells were seeded in 96-well plates at a concentration of 3000 cells per well, with three replicates for each treatment. The plates were then placed in the Incucyte SX5 for imaging at predetermined time intervals. Subsequently, a 10% solution of CCK8 in complete medium was added to the cells at designated time points and incubated for 1 h. The absorbance was determined at 450 nm using SpecteaMax iD5 (Molecular Devices, Shanghai).

### EdU staining

A total of 20,000 cells were plated in 24-well plates and permitted to adapt for an overnight period. Following this, cells were exposed to 10 mM 5-ethynyl 2′-deoxyuridine (EdU) for 2 h. Subsequently, they were fixed with 4% paraformaldehyde for 15 min and permeabilized with 0.3% Triton X-100 for 10 min. Blocking was performed using 5% bovine serum albumin for 1 h. Cells then underwent a 30-min incubation with Click reaction cocktails. Finally, nuclei were stained with 4′,6-diamidino-2-phenylindole (DAPI) at room temperature for 30 min before microscopic examination.

### Flow cytometry

Following a 48-h treatment with either CUDC-907 or dimethylsulfoxide (DMSO), the cells were harvested, centrifuged, and subsequently resuspended in a phosphate-buffered saline (PBS) solution. For cell cycle analysis, cells were fixed in 75% ethanol for a duration of 24 h, followed by staining with 1 μL of propidium iodide and 1 μL of RNase in 300 μL of PBS for each sample. A CytoFLEX Flow Cytometer (Beckman, Florida, USA) was utilized for the analysis, with each experimental group comprising three replicates. To assess apoptosis, cells were stained with FITC-annexin V and propidium iodide for 30 min before flow cytometric analysis.

### Western blotting

Cellular proteins were extracted using RIPA buffer with 1% phenylmethanesulfonyl fluoride (PMSF) and phosphatase inhibitors. Post-lysis, samples were centrifuged, and protein concentrations were quantified using the bicinchoninic acid (BCA) protein assay kit. Equal amounts were denatured, resolved via SDS-PAGE, and transferred to polyvinylidene fluoride membranes (Millipore, Massachusetts, USA). The membranes were blocked with 5% bovine serum albumin and probed with specific antibodies. The signals were visualized employing EZ ECL pico (Life-ilab, AP34L024) and detected with a FUSION FX EDGE system (Vilber, PAR, France).

### Comet assay

Comet assays were conducted in accordance with the manufacturer’s guidelines (Beyotime, C2041S). GBM cells were treated with TMZ and CUDC-907 for 48 h, and then trypsinized and resuspended in 1 mL of culture medium. The cells were combined with 0.7% low-melting-point agarose and lysed with lysis buffer at 4 °C for 2 h. Following this, they underwent horizontal electrophoresis for 30 min in an alkaline buffer (200 mM NaOH, 1 mM EDTA) and were stained with propidium iodide. Comet tails were examined under a fluorescence microscope (Olympus, BX53, Tokyo, Japan), with the length of the tails serving as an indicator of DNA damage.

### Clone formation assay

Cells were plated in 6-well plates at a density of 1000 cells per well. After 72 h of treatment, they were maintained in a drug-free medium for two weeks. Colonies were washed with PBS and fixed in 4% paraformaldehyde. Subsequently, colonies were stained with crystal violet for 20 min and rinsed with tap water. Once air-dried, crystal violet was dissolved using absolute ethanol, and the absorbance was measured.

### Immunofluorescence staining

After a treatment period of 7 days, brain organoids and glioblastoma organoids (GBOs) were harvested, dehydrated, and prepared as frozen sections that were subsequently stored at −80 °C. For the immunofluorescence staining procedure, antigen retrieval was achieved using sodium citrate, followed by a blocking step with bovine serum albumin for 1 h. Primary antibodies were allowed to incubate at 4 °C overnight, while secondary antibodies were applied at room temperature for 1 h. DAPI was used for staining nuclei. The samples were then examined under a confocal microscope (ZEISS, LSM980, Baden-Württemberg, Germany).

### Immunohistochemistry

In the immunohistochemical staining process, paraffin-embedded tumor specimens were cut into slices that were 5 μm thick, followed by deparaffinization and rehydration. Antigen retrieval was performed by heating the sections in a microwave for 20 min in a 10 mM citrate buffer (pH 6.0). Following the inhibition of endogenous peroxidase activity and subsequent blocking with goat serum, the sections were sequentially incubated with primary antibodies and a secondary antibody conjugated to horseradish peroxidase. Staining visualization was conducted using diaminobenzidine, and the sections were then counterstained with hematoxylin. Finally, the stained sections were analyzed using a microscope.

### Migration and wound healing assay

For the migration assay, 24-well Boyden chambers with an 8 μm pore size (Corning, Beijing, China) were employed. Cells suspended in serum-free media were added to the upper chamber, while the lower chamber contained media supplemented with 10% fetal bovine serum as a chemotactic agent. Following a 5-h incubation period, the cells were fixed using 4% paraformaldehyde and subsequently stained with crystal violet. The average number of cells was calculated from randomly chosen microscopic fields, analyzing three fields per filter. In the wound healing assay, cells suspended in serum-free media were seeded in 24-well plates and allowed to grow until they reached full confluence. Wounds were created with a 10 μL pipette tip, and the distances migrated were observed under a microscope.

### Hematoxylin-eosin staining

The tissue samples underwent a series of steps, including fixation, dehydration, embedding, and sectioning. The slides were subjected to staining following the protocol provided by the hematoxylin-eosin staining kit (Beyotime, C0105S).

### *In vivo* study

All experimental protocols for animal studies received approval from the Jinfeng Laboratory Animal Center. NOD-SCID female mice, aged between 4 and 6 weeks, were sourced from GemPharmatech (Chengdu, China). LN229 cells labeled with firefly luciferase were injected intracranially at a density of 2 × 10^5^ cells in 5 μL of PBS. Tumor growth was monitored through bioluminescence imaging using an *in vivo* imaging system (PerkinElmer, Waltham, Massachusetts, USA) and analyzed with a living image software for the *in vivo* imaging system (PerkinElmer). Following the verification of tumor presence, the mice with tumors were allocated into four distinct treatment groups for oral administration (*n* = 3 per group): a solvent control group, a TMZ group (66 mg/kg administered five times weekly), a CUDC-907 group (100 mg/kg administered five times weekly), and a combination group receiving both TMZ and CUDC-907.^28^^,^^29^ All drug preparation protocols were carefully adhered to according to the manufacturer’s instructions. After 2 weeks of treatment, the brains of the mice were collected and subsequently embedded in paraffin for immunohistochemistry and hematoxylin-eosin staining.

### Cerebral organoid and GBO culturing

Cerebral organoids and GBOs were cultured as previously described.^30^^,^^31^ Once the human embryonic stem cells are stably growing, they are dissociated using TrypLE (Thermo Fisher Scientific cat. no.12604013) and cultured in human embryonic stem cell induction medium (EIM) containing the ROCK inhibitor Y27632 (50 μM, Merck cat. no. 688000-5) and basic fibroblast growth factor (bFGF) (4 ng/mL, Peprotech, cat. no. 100-18B) to form embryoid bodies in low-attachment 96-well U-bottom plates (day 0). On day 4, the medium is replaced with EIM without bFGF/ROCK inhibitor. On day 6, the medium is switched to neural induction medium (NIM), with medium changes every two days. Upon significant neuroepithelial formation (day 12), embryoid bodies are transferred to pre-cooled Parafilm templates and gently embedded into pre-melted 4 °C Matrigel droplets (approximately 30 μL/embryoid body, Corning, cat. no. 356234), and cultured in cerebral organoid differentiation medium (CODM), with medium change after 48 h. On day 16, the medium was changed to cerebral organoid maintenance medium (COMM), and the cultures were transferred to an orbital shaker installed in an incubator (37 °C, 80 rpm), with medium changes every other day for maintenance. Drug treatment is initiated after day 28. The culture process for patient-derived tumor organoids involves transporting the tissue in preservation solution, followed by three washes with PBS containing 1% penicillin-streptomycin and 1% amphotericin B. The tissue is then mechanically minced into 1 mm^3^ fragments in fresh, cold preservation solution. After three additional PBS washes, red blood cells are lysed for 15 min, followed by three washes with DMEM/F12. The fragments are then suspended in culture medium and transferred to an orbital shaker installed in an incubator (37 °C, 5% CO_2_, 120 rpm). After two weeks of cultivation, drug treatment is applied to evaluate therapeutic response. The working concentration of CUDC-907 is 200 nM, determined based on the dosage administered to animals.

### Patient data analysis

Patient and gene expression datasets were acquired from the R2 genomics analysis and visualization platform (http://hgserver1.amc.nl/cgi-bin/r2/main.cgi). The Kaplan–Meier survival analysis was conducted using the expression cutoff determined by the R2 algorithm, distinguishing between high and low expression levels. *P*-values from the log-rank test were sourced from the platform.

### Statistical analysis

Statistical analyses were conducted utilizing GraphPad Prism software. A two-tailed Student’s *t*-test was utilized to assess statistical significance at a confidence level of 95%. A *P*-value < 0.05 was deemed statistically significant, with ∗*P* < 0.05, ∗∗*P* < 0.01, and ∗∗∗*P* < 0.001 reflecting varying degrees of statistical significance as presented in the figures. Survival analyses were executed using the Kaplan–Meier method.

## Results

### CUDC-907 inhibits the growth of GBM without noticeable toxicity

**Figure 1:**
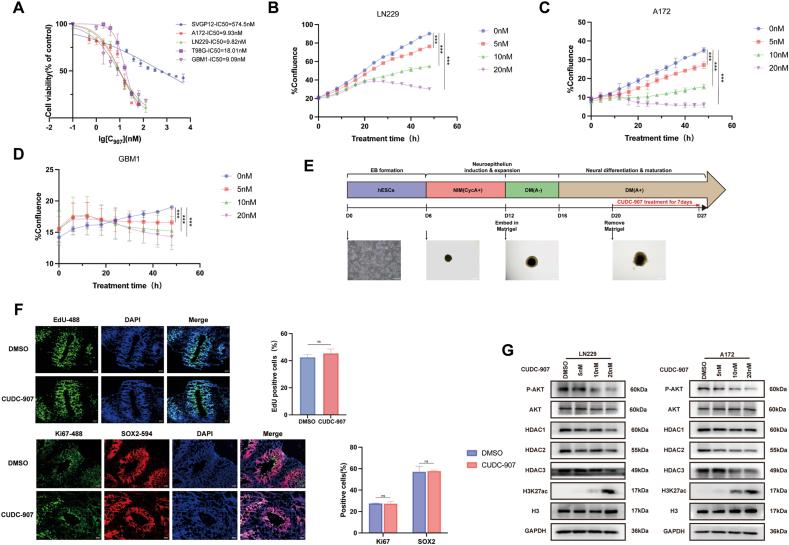
CUDC-907 inhibits the growth of glioblastoma without noticeable toxicity. **(A)** Dose–response curves for CUDC-907 in the four glioblastoma cell lines and SVG p12. Cell viability was measured after 48 h of treatment. **(B**–**D)** Viability of LN229, A172, and GBM1 cells after treatment with 5, 10, and 20 nM CUDC-907. DMSO was used as a control. **(E)** Overview and representative diagram of the human cerebral organoid cultivation process. Scale bars = 500 μm. **(F)** EdU and immunofluorescence results after 7-day treatment of human cerebral organoids with CUDC-907. Scale bars = 50 μm. **(G)** Immunoblot analyses of downstream elements after CUDC-907 (5, 10, and 20 nM), for either 24 h for AKT and p-AKT, or 48 h for HDAC1, HDAC2, HDAC3, H3, and H3Ac. The data were expressed as mean ± standard deviation. Student’s *t*-test was performed to analyze significance. ∗*P* < 0.05, ∗∗*P* < 0.01, and ∗∗∗*P* < 0.001.

To evaluate the anti-tumor activity of CUDC-907, we conducted experiments on three GBM cell lines, LN229, A172, and T98G, and one primary cell line, GBM1. The GBM cell lines were treated with varying concentrations of CUDC-907 for 48 h. Compared with the SVG p12, a human brain astrocyte cell line, the GBM cell lines are more sensitive to CUDC-907. The median inhibition concentration (IC50) observed across GBM cell lines ranged from 9.09 nmol/L to 18.01 nmol/L, while it in SVG p12 was 574.5 nmol/L (Fig. 1A). The viability of cells was measured by Incucyte assay using the integrated confluence algorithm as a surrogate for cell numbers. Using three different concentrations of CUDC-907 to treat LN229, A172, and GBM1, the Incucyte assay results showed that CUDC-907 significantly inhibited the growth of those GBM cells, and the inhibition showed remarkable dose dependency (Fig. 1B–D).

Given that the primary objective of the drug test is to provide insights for clinical application, evaluating drug safety is crucial. We conducted validations at both cellular and organoid levels. As the result showed in Figure 1A. Human brain astrocyte cell line SVG p12 was tested as normal cell control, which IC50 was found to be 574.5 nmol/L, approximately 50 times higher than the IC50 for GBM cells (Fig. 1A). To more accurately simulate the potential toxicity of CUDC-907 to normal brain tissue *in vivo*, we followed the method of Madeline A. Lancaster to culture human cerebral organoids by inducing the differentiation of human embryonic stem cells *in vitro* to culture human cerebral organoids (Fig. 1E).^30^ After treating these organoids with CUDC-907 for 7 days, we conducted EdU assays and immunofluorescence staining experiments (Fig. 1F). We found that the EdU staining ratio is not significantly different between DMSO and CUDC-907-treated cerebral organoid tissues, which are all about 40%. The expression of proliferation marker Ki67 and stem cell marker SOX2 also showed similar results, which are all about 27% and 57%. The results demonstrated that CUDC-907 had no significant effect on either the proliferation or pluripotency of the human brain organoids.

We confirmed the dual inhibition of the HDAC and PI3K pathways by CUDC-907 by measuring the levels of acetylated proteins (histones) as markers of HDAC inhibition and phosphorylated proteins downstream of PI3K (p-AKT) in LN229 and A172 cells. Results showed p-AKT, HDAC1, HDAC2, and HDAC3 demonstrated dose-dependent down-regulation in response to CUDC-907 treatment, while the acetylation of histone was up-regulated (Fig. 1G). These results indicated that CUDC-907 inhibits cell growth via its classic targets PI3K and HDACs in GBM without noticeable toxicity.

### CUDC-907 regulates the cell cycle of GBM cells by inhibiting MYC expression to suppress GBM cell proliferation

**Figure 2:**
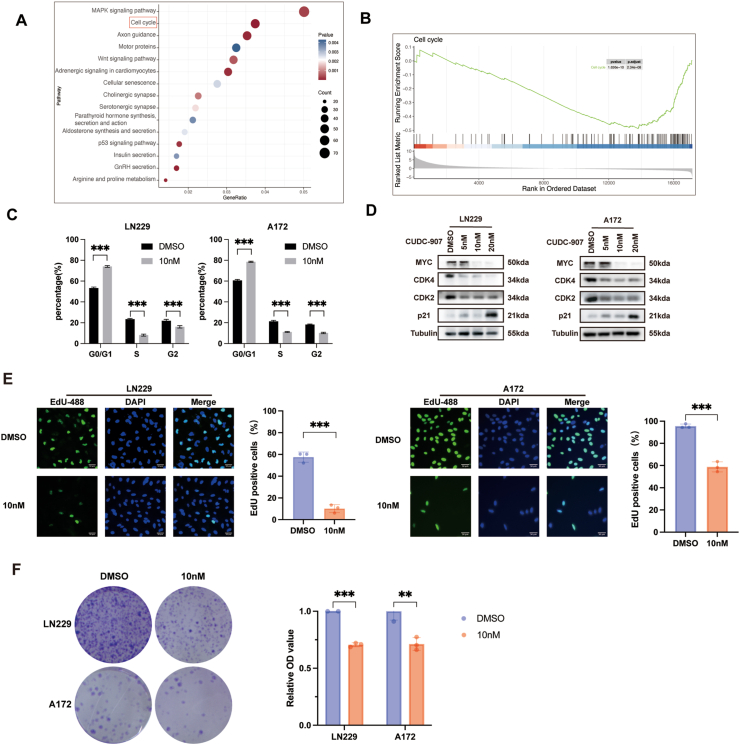
CUDC-907 regulates the cell cycle of glioblastoma cells by inhibiting MYC expression to suppress glioblastoma cell proliferation. **(A)** RNA sequencing analysis was conducted on LN229 cells exposed to 10 nM CUDC-907 or DMSO for 24 h. The KEGG enrichment analysis of total differential genes. **(B)** The cell cycle was examined using Gene Set Enrichment Analysis (GSEA) based on the differentially expressed genes following CUDC-907 treatment in LN229 cells. The *P* value was calculated with the GSEA software. **(C)** Cell cycles of both cell lines were arrested in the G1 phase after 48 h of treatment with CUDC-907. **(D)** The levels of CDK2, CDK4, p21, and MYC were assessed by Western blotting in LN229 and A172 following 48 h of CUDC-907 exposure. DMSO was used as a control. **(E)** EdU-positive LN229 and A172 cells were identified following treatment with CUDC-907 (10 nM) or DMSO for 48 h. Scale bars = 50 μm. **(F)** The impact of indicated concentrations of CUDC-907 or DMSO on the colony formation capabilities of LN229 and A172. The data were expressed as mean ± standard deviation. Student’s *t*-test was performed to analyze significance. ∗*P* < 0.05, ∗∗*P* < 0.01, and ∗∗∗*P* < 0.001.

To gain deeper insights into the mechanism by which CUDC-907 inhibits cell growth in GBM, we conducted transcriptome sequencing (RNA sequencing) on cells treated with CUDC-907 for 24 h, alongside a control group of cells treated with DMSO. Kyoto Encyclopedia of Genes and Genomes (KEGG) enrichment analysis of the differentially expressed genes revealed marked alterations in pathways associated with the cell cycle (Fig. 2A). Further, we performed Gene Set Enrichment Analysis (GSEA) on these genes, and our findings indicated a significant down-regulation of cell cycle-related genes in comparison to the control group (Fig. 2B). These results imply that CUDC-907 may influence the regulatory mechanisms governing the cell cycle in GBM cells.

Therefore, to confirm the results, we conducted flow cytometry analysis on CUDC-907 treated GBM cell lines LN229 and A172 and found that the cell cycles of both cell lines were arrested in the G1 phase, the proportion of cell in G0/G1 phase increased from 52% to 74% in LN229, while it increased from 60% to 78% in A172 (Fig. 2C). Given the critical role of cyclin-dependent kinase inhibitor 1A (CDKN1A)/p21 in the regulation of G1 cell cycle arrest,^32^ and considering that the MYC gene can inhibit the transcription of p21 by suppressing its promoter,^33^^,^^34^ while CUDC-907 inhibits HDAC and PI3K, thereby inhibiting MYC through transcriptional and post-translational mechanisms,^35^ we assessed the levels of MYC, p21, and G1/S cell cycle checkpoint-related proteins following CUDC-907 treatment. Western blotting results showed CUDC-907 down-regulates MYC, leading to increased p21 expression, which in turn regulates the cell cycle arrest of GBM cells in the G1 phase (Fig. 2D). Furthermore, to investigate the effect of CUDC-907 on the proliferation of GBM cells, we performed EdU assays and colony formation assays. The results demonstrated that the proliferation and colony formation capabilities of GBM cells were significantly inhibited after CUDC-907 treatment (Fig. 2E and F).

### CUDC-907 inhibits the migratory and invasive capabilities of GBM cells and promotes apoptosis

**Figure 3:**
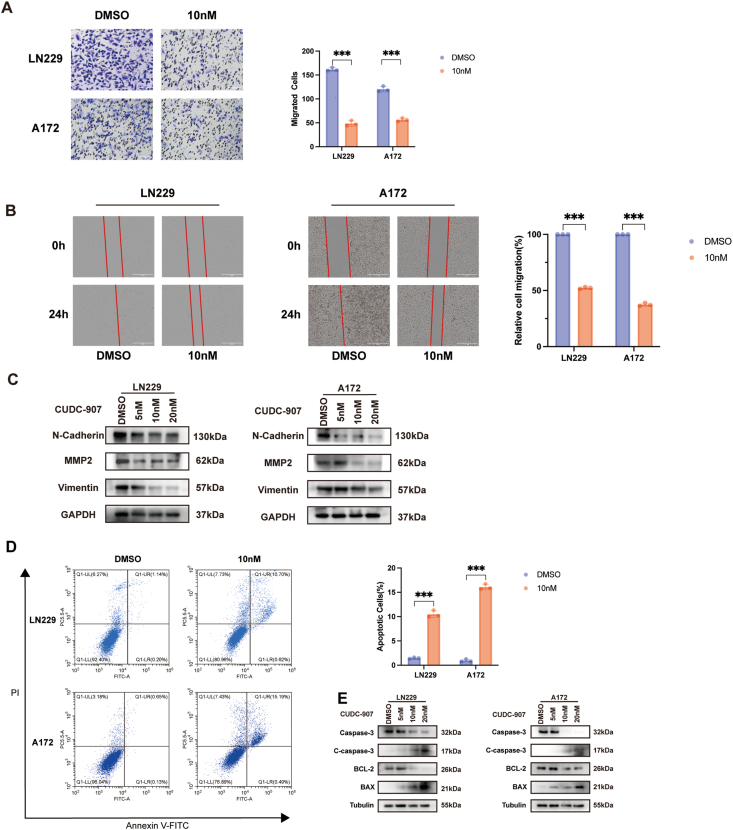
CUDC-907 inhibits the migratory and invasive capabilities of glioblastoma cells and promotes apoptosis. **(A)** Migration assay was performed after treatment with CUDC-907 (10 nM) or DMSO for 48 h. Scale bars = 100 μm. **(B)** Wound healing assay was performed with 24 h treatment of CUDC-907 (10 nM) or DMSO. Scale bars = 1 mm. **(C)** The levels of N-Cadherin, MMP2, and Vimentin were assessed by Western blotting in LN229 and A172 following 48 h CUDC-907 (5, 10, and 20 nM) or DMSO exposure. **(D)** Flow cytometry assessed the apoptosis ratio in LN229 and A172 treated with CUDC-907 (10 nM) or DMSO for 48 h. **(E)** The levels of Bcl-2, BAX, caspase-3, and cleaved caspase-3 were assessed by Western blotting in LN229 and A172 following 48 h of CUDC-907 (5, 10, and 20 nM) or DMSO exposure. The data were expressed as mean ± standard deviation. Student’s *t*-test was performed to analyze significance. ∗*P* < 0.05, ∗∗*P* < 0.01, and ∗∗∗*P* < 0.001.

Migration and invasion are critical characteristics that enable GBM to sustain its aggressive phenotype. To explore the impact of CUDC-907 on the migratory and invasive properties of GBM cells, we treated the cells with CUDC-907 for 24 h, followed by cell counting and plating. Our results demonstrated a significant reduction in both the migration ability and speed of GBM cells upon CUDC-907 treatment (Fig. 3A and B). Enhanced migratory capacity is often associated with tumor metastasis, where the epithelial–mesenchymal transition plays a pivotal role. During epithelial–mesenchymal transition, epithelial cells adopt mesenchymal traits, thereby increasing their motility and capacity for migration. Consequently, we assessed the expression levels of epithelial–mesenchymal transition-related proteins and found that treatment with CUDC-907 led to a decrease in N-cadherin and Vimentin expression in the treated cells. The levels of matrix metalloproteinase-2 (MMP2) also decreased following CUDC-907 treatment, indicating that the invasive capability of GBM is inhibited by CUDC-907 (Fig. 3C).

Furthermore, we explored whether CUDC-907 induces apoptosis in GBM cells. Cells treated with CUDC-907 were harvested for annexin V/propidium iodide staining followed by flow cytometry analysis. The treatment with CUDC-907 led to a notable increase in the population of Annexin V-positive cells (Fig. 3D), suggesting that CUDC-907 effectively induces apoptosis in these cell lines. To investigate the pathway through which CUDC-907 induces apoptosis, we examined apoptosis-related proteins (Fig. 3E).^36^^,^^37^ The results showed that CUDC-907 disrupts the balance between Bcl-2 and Bax and activates caspase-3 cleavage, thereby inducing apoptosis in GBM cells.

### Enhanced inhibitory effect on GBM by the combined use of CUDC-907 and TMZ

**Figure 4:**
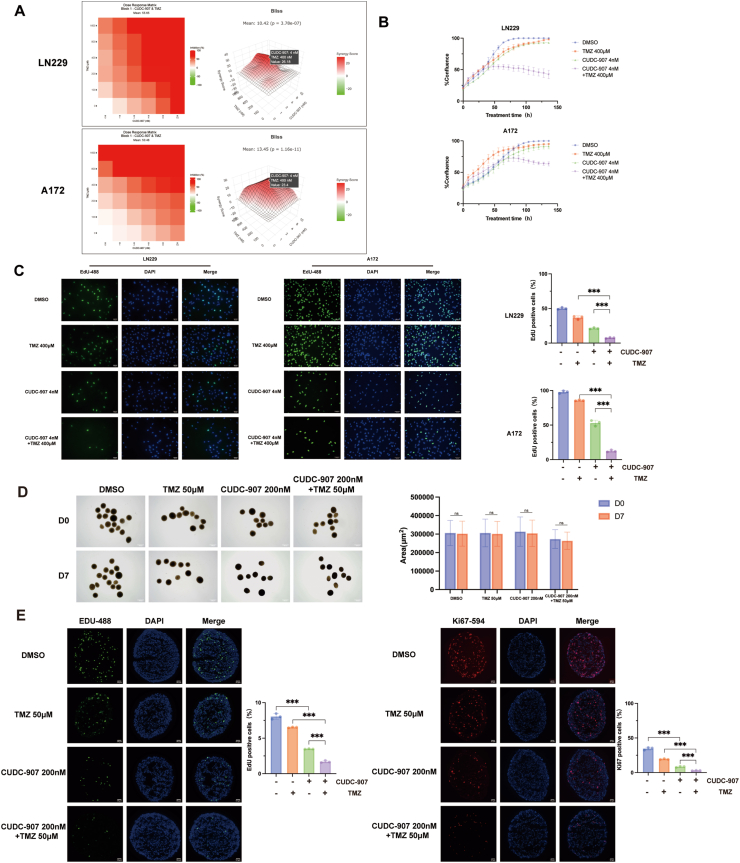
Enhanced inhibitory effect on glioblastoma by combined use of CUDC-907 and temozolomide (TMZ). **(A)** 2D heatmaps show the percentage of cell viability after different doses of CUDC-907 and TMZ treatment in LN229 and A172. Cell viability was measured by the CCK8 assay after 48 h of drug treatment. The SynergyFinder Plus online tool was used for Bliss synergistic analysis to evaluate the synergistic effect of the combination treatment in LN229 and A172. **(B)** Incucyte cell confluence assays show the synergistic effect of the CUDC-907 and TMZ treatment on cell proliferation (% confluence) over time. **(C)** EdU positive LN229 and A172 cells after treatment with CUDC-907 and TMZ for 48 h. Scale bars = 50 μm. **(D)** Representative diagram of GBO with treatment of CUDC-907 and TMZ for 7 days. Scale bars = 500 μm. **(E)** EdU and Ki67 positive cells in GBO after treatment with CUDC-907 and TMZ for 7 days. Scale bars = 50 μm. The data were expressed as mean ± standard deviation. Student’s *t*-test was performed to analyze significance. ∗*P* < 0.05, ∗∗*P* < 0.01, and ∗∗∗*P* < 0.001.

**Figure S2:**
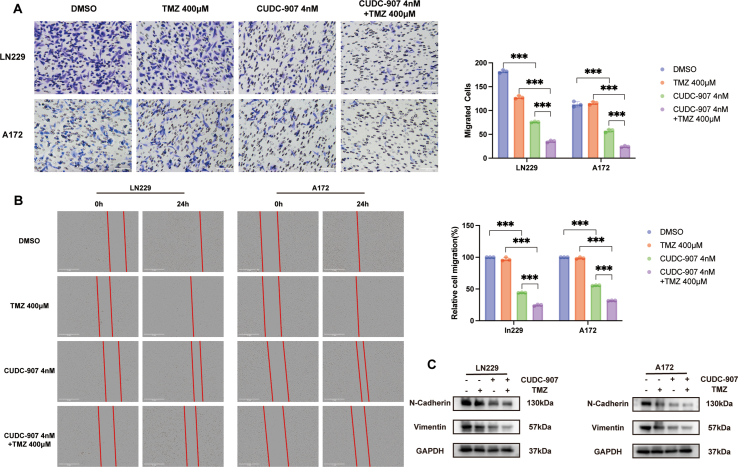
Combination of CUDC-907 and temozolomide (TMZ) inhibits the migratory and invasive capabilities of glioblastoma cells. **(A)** Migration assay was performed after treatment with CUDC-907 and TMZ for 48 h. DMSO was used as a control. Scale bars = 100 μm. **(B)** Wound healing assay was performed with 24 h treatment of CUDC-907 and TMZ for 48 h. DMSO was used as a control. Scale bars = 1 mm. **(C)** The levels of N-Cadherin and Vimentin were assessed by Western blotting in LN229 and A172 cells following 48 h exposure to CUDC-907 and TMZ. DMSO was used as a control. The data were expressed as mean ± standard deviation. Student’s *t*-test was performed to analyze significance. ∗*P* < 0.05, ∗∗*P* < 0.01, and ∗∗∗*P* < 0.001.

Given that TMZ resistance is a clinically meaningful and substantial obstacle that must be overcome to achieve successful treatment outcomes for GBM, we performed assays to determine if CUDC-907 and TMZ have synergistic effects on the inhibition of GBM cells. First, we observed that the combined use of CUDC-907 and TMZ in LN229 and A172 cells synergistically reduced the number of viable GBM cells. SynergyFinder Plus online tool was used to study the synergistic effect of the combination treatment of GBM cells *in vitro*.^38^ The average Bliss synergy scores across different dose ranges were all greater than 10 (Fig. 4A). Cell confluence assays confirmed the synergistic effects of CUDC-907 plus TMZ treatment on cell proliferation in both in LN229 and A172, compared with the cells treated with CUDC-907 or TMZ alone, cell growth was significantly inhibited in the treated cells with the compound combination (Fig. 4B). Similarly, the EdU assay revealed that GBM cell proliferation was significantly suppressed following the combined treatment both in LN229 and A172 (Fig. 4C). Additionally, the dual drug combination inhibited the migratory and invasive capabilities of GBM cells (Fig. S2A–S2C).

In addition, we conducted experiments on GBOs. The GBOs were treated with CUDC-907 and TMZ, both separately and in combination, for one week. The concentration of CUDC-907 was calculated based on *in vivo* experimental concentrations, while TMZ was used at a concentration of 50 μM.^31^ After one week, the volume of the GBOs showed an insignificant change (Fig. 4D). However, EdU and immunofluorescence staining performed after sample collection revealed that the proliferation of GBOs was significantly inhibited following the combined treatment (Fig. 4E). GBOs effectively retain the tumor’s characteristics to a high degree, providing an improved means for evaluating the efficacy of the combined CUDC-907 and TMZ treatment. Therefore, our results demonstrate that CUDC-907 combined with TMZ has remarkable synergistic effects on anti-tumorigenesis in GBM.

### CUDC-907 enhances its synergy with TMZ by inhibiting DNA damage repair

**Figure 5:**
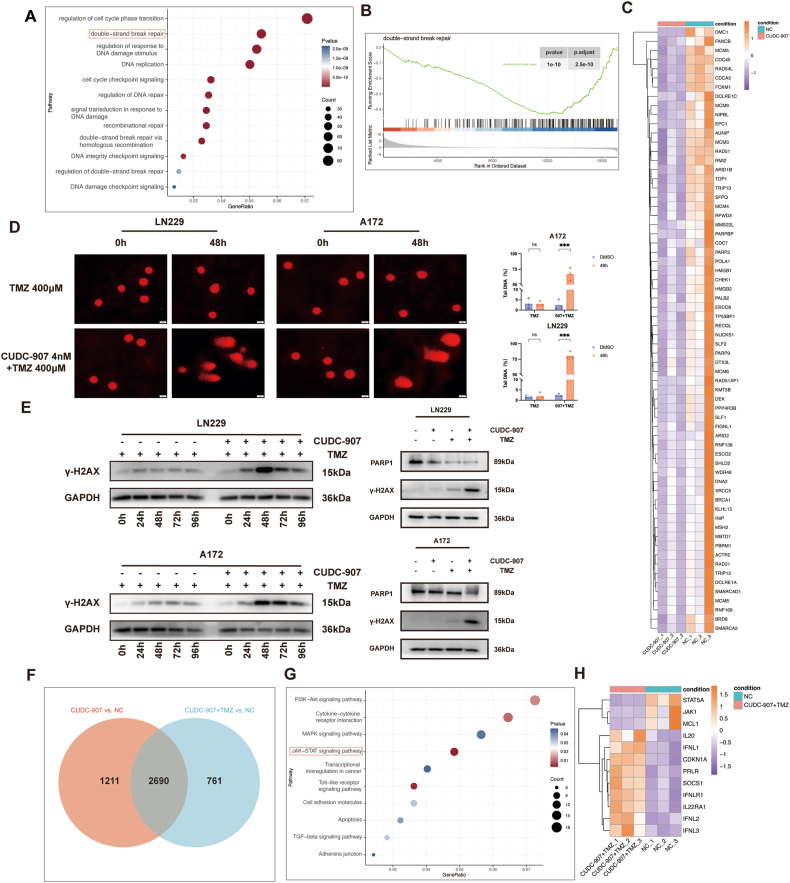
CUDC-907 enhances its synergy with temozolomide (TMZ) by inhibiting DNA damage repair. **(A)** RNA sequencing analysis of LN229 cells treated with 10 nM CUDC-907 and DMSO for 24 h. The Gene Ontology (GO) enrichment analysis of down-regulated genes. **(B)** Double-strand break repair was analyzed with GSEA according to down-regulated genes after CUDC-907 treatment in LN229 cells. The *P* value was calculated with the GSEA software. **(C)** Heatmap of gene expression levels about the double-strand break repair signaling pathway in down-regulated genes after CUDC-907 treatment. **(D)** Representative pictures of comet assays after treatment with CUDC-907 and TMZ. Scale bars = 50 μm. **(E)** The levels of γ-H2AX over time (E, left), and of γ-H2AX and PARP were assessed by Western blotting following exposure with CUDC-907 and TMZ for 48 h (E, right). **(F, G)** 761 genes were identified by the total differential set after CUDC-907 treatment, and the total differential set after CUDC-907 and TMZ treatment gene set. The KEGG enrichment analysis of the 761 genes. **(H)** Heatmap of gene expression levels about the JAK-STAT signaling pathway in down-regulated genes after CUDC-907 treatment. The data were expressed as mean ± standard deviation. Student’s *t*-test was performed to analyze significance. ∗*P* < 0.05, ∗∗*P* < 0.01, and ∗∗∗*P* < 0.001.

Our previous work demonstrated that combining CUDC-907 with TMZ significantly inhibits the growth of GBM cells compared with using either compound alone. TMZ is stable at acidic pH but becomes activated at physiological pH by converting into the metabolite 5-(3-methyltriazen-1-yl) imidazole-4-carboxamide (MTIC). Subsequently, MTIC hydrolyzes to produce methyl diazonium ions, which are electrophilic methylating agents that cause DNA damage. The negatively charged DNA acts as a nucleophile, and the methyl group from the methyl diazonium ion is transferred to the DNA, leading to the formation of multiple DNA adducts. This creates opportunities for mismatched base pairing, ultimately resulting in cytotoxicity.^39^ We conducted RNA sequencing on cells treated with CUDC-907 for 24 h and the control group cells. Gene Ontology (GO) enrichment analysis of all genes down-regulated by CUDC-907 treatment revealed significant changes in pathways related to DNA double-strand break repair (Fig. 5A). Subsequent GSEA and heatmap visualization of gene expression changes showed a marked down-regulation of genes associated with DNA double-strand break repair, including RAD and PARP families (Fig. 5B and C). We hypothesize that after TMZ induces DNA damage through methylation, the DNA damage repair pathways in the cells are inhibited by CUDC-907, which is a potential mechanism for the synergistic effect between CUDC-907 and TMZ. This hypothesis is supported by comet assay results and the detection of the DNA damage marker γ-H2AX^40^ (Fig. 5D and E). After treating the cells with the drug for 24 h, 48 h, 72 h, and 96 h, we conducted Western blotting assays. The results showed that the combination of CUDC-907 and TMZ significantly up-regulated γ-H2AX compared with TMZ alone, with the strongest effect observed at 48 h. Subsequently, we examined the expression of the DNA repair-related protein PARP1 after 48 h of treatment. The results indicated that PARP1 expression was significantly inhibited when both drugs were used together. These results suggest that CUDC-907 may inhibit the expression of DNA damage repair-related genes, thereby enhancing the DNA damage induced by TMZ in GBM cells. This effect further suppresses cell growth and promotes cell death.

Next, we performed RNA sequencing analysis on cells treated with the combination of CUDC-907 and TMZ, as well as on control group cells. After excluding genes that showed changes similar to those observed with CUDC-907 monotherapy, we identified 761 genes that were altered by the combined treatment of CUDC-907 and TMZ (Fig. 5F). KEGG enrichment analysis of these 761 genes revealed significant changes in the JAK/STAT pathway, which is closely related to TMZ resistance^39^ (Fig. 5G). The heatmap of gene expression changes in this pathway showed significant variations in the expression levels of related genes (Fig. 5H), providing a new perspective on the mechanism by which CUDC-907 reduces TMZ resistance and enhances TMZ sensitivity.

### Both CUDC-907 monotherapy and its combination with TMZ can effectively inhibit the growth of GBM *in vivo*

**Figure 6:**
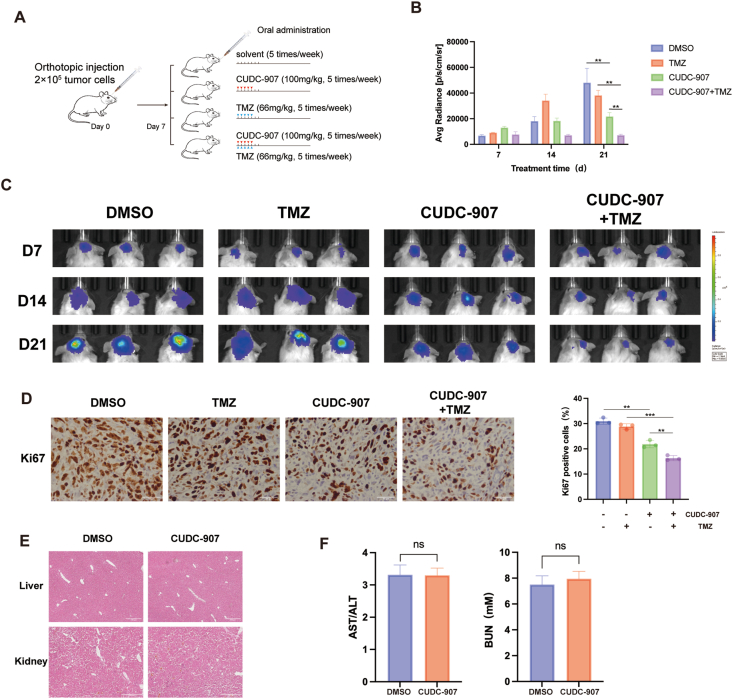
Both CUDC-907 monotherapy and its combination with temozolomide can effectively inhibit the growth of glioblastoma *in vivo*. **(A)** Orthotopic xenografts were established by intracranially injecting LN229 cells (luciferase-expressing) into NOD-SCID mice. **(B, C)** Representative bioluminescence images and quantification of intracranial xenografts derived from LN229-luc cells treated with solvent/drugs. **(D)** Immunohistochemistry staining images of Ki67 in xenograft tumors in the mouse brains. Scale bars = 50 μm. **(E)** Representative hematoxylin-and-eosin-stained images of the liver and kidney tissues from mice treated with CUDC-907 or DMSO. Scale bars = 500 μm. **(F)** Serum levels of AST/ALT and BUN in mice treated with CUDC-907 or DMSO. The data were expressed as mean ± standard deviation. Student’s *t*-test was performed to analyze significance. ∗*P* < 0.05, ∗∗*P* < 0.01, and ∗∗∗*P* < 0.001.

Evaluating the synergistic inhibitory effect in animal models is crucial. We established an orthotopic xenograft model by intracranially injecting LN229 (luciferase-expressing) cells into NOD-SCID mice. One week after tumor implantation, we established four treatment groups: a solvent control, CUDC-907 (100 mg/kg, administered five times a week), TMZ (66 mg/kg, administered five times a week), and a combination treatment group of CUDC-907 and TMZ (Fig. 6A). *In vivo* bioluminescence imaging, along with hematoxylin-eosin staining, revealed that both CUDC-907 and TMZ effectively inhibited tumor growth; however, the combination treatment exhibited the highest efficacy, corroborating our *in vitro* results (Fig. 6B and C). Additionally, immunohistochemistry staining confirmed that the combination treatment significantly reduced the expression of the proliferation marker Ki67 in the tumors (Fig. 6D). These data demonstrate the potential application of CUDC-907 in combination with TMZ for the oral treatment of GBM. Furthermore, hematoxylin-eosin staining of liver and kidney tissues following drug treatment revealed no structural abnormalities in these organs, and there were no significant differences observed in the functional indicators of liver and kidney activity, further supporting the *in vivo* safety profile of CUDC-907 (Fig. 6E and F).

## Discussion

GBM, recognized as the most common primary brain tumor, presents significant challenges in treatment. Despite advancements in therapeutic approaches in recent years, the prognosis for patients remains concerning. This tumor is characterized by rapid growth and high invasiveness, which severely limit the effectiveness of surgical resection, radiotherapy, and chemotherapy, resulting in a notably high rate of recurrence. Therefore, the exploration of new treatment strategies has become critically important, aimed at improving patient survival rates and quality of life. Currently, researchers are focused on developing novel therapies such as targeted treatments and immunotherapies,^41^ delving into the molecular mechanisms underlying GBM in the hope of identifying more effective treatment options to address this significant medical challenge.

The findings confirm that CUDC-907 effectively inhibits the growth, proliferation, and migratory invasion capabilities of GBM, leading to cell cycle arrest through the down-regulation of MYC expression and promotion of apoptosis. Notably, CUDC-907 exhibits minimal toxicity to normal brain cells and tissues. Similar observations have been reported in studies involving other types of tumors.

The treatment of GBM is notably complicated by the development of resistance to TMZ. Therefore, we investigated whether CUDC-907 could enhance the drug sensitivity of GBM to TMZ. Through *in vitro* and *in vivo* experiments, we found that the combination of CUDC-907 and TMZ significantly improved the inhibitory effects on GBM. The synergistic effect of CUDC-907 and TMZ supports the development of clinical trials for low-dose combinations to reduce toxicity. In pediatric high-grade gliomas, CUDC-907 enhances sensitivity to radiation by inhibiting DNA repair mechanisms.^29^ This supports our findings that CUDC-907 down-regulates genes associated with DNA repair, inhibits DNA repair pathways, and reduces the activation of TMZ-induced DNA repair systems.

The blood–brain barrier is a critical consideration in the development of effective medications for brain tumors. Currently, a clinical trial (NCT03893487) is underway with the primary objective of confirming the penetration of CUDC-907 through the blood–brain barrier in children and young individuals newly diagnosed with diffuse intrinsic pontine glioma, recurrent medulloblastoma, or recurrent high-grade glioma by measuring the concentrations of CUDC-907 and its metabolites in primary tumor tissues. These are favorable supports for the future clinical application of CUDC-907 in the treatment of GBM.

Compared with existing related studies, this research introduces brain organoids and GBOs, which better preserve the structure and microenvironment of normal or tumor tissues. GBOs can retain native cell–cell interactions and recapitulate the heterogeneity of their corresponding parental tumors, including histology, immunohistology, transcriptomic signatures, and cell populations.^31^ This allows for a more accurate simulation of the human body’s response to drugs. GBOs overcome the limitations of simplified microenvironments in cell lines and the lengthy modeling cycle of patient-derived xenografts, providing a high-fidelity platform for preclinical efficacy evaluation.

In conclusion, the use of CUDC-907, whether as a monotherapy or in combination with TMZ, has demonstrated promising efficacy at the cellular, organoid, and animal levels. We hope these findings offer new insights into the treatment of GBM, the highly malignant tumor, and potentially pave the way for more effective therapeutic strategies.

## CRediT authorship contribution statement

**Chencheng Fang:** Writing – review & editing, Writing – original draft, Software, Methodology, Investigation, Conceptualization. **Pan Gou:** Methodology, Formal analysis, Data curation. **Dandan Zhang:** Resources, Methodology, Conceptualization. **Xuanxuan Wu:** Funding acquisition. **Xiao Li:** Software, Methodology. **Man Li:** Investigation, Formal analysis. **Lu Gan:** Methodology, Investigation. **Jinjin Luo:** Methodology, Investigation. **Hongjuan Cui:** Visualization, Validation. **Man Xu:** Writing – review & editing, Visualization, Methodology, Conceptualization. **Ping Liang:** Supervision, Conceptualization.

## Ethics declaration

This study was conducted in compliance with the principles of the Declaration of Helsinki. Informed consent was obtained from all the subjects. Ethics approval for human subjects was provided by the Ethics Committee of the Children’s Hospital of Chongqing Medical University (ethics number: 2024-311). Ethics approval for animal work was provided by the Jinfeng Laboratory Institutional Animal Care and Use Committee.

## Data availability

All data and materials in this paper are available upon reasonable request.

## Funding

This work was supported by the Chongqing Municipal Health Commission Medical Research Project (China) (No. 2024WSJK028) and Chongqing Municipal Education Commission Science and Technology Research Project (China) (No. KJON202300425).

## Conflict of interests

The authors declared no potential conflict of interests.
