# Supplementary material for: CUDC-907 inhibits glioblastoma and enhances glioblastoma sensitivity to temozolomide by inhibiting DNA damage repair
Source: Genes Dis. 2025 Nov 24;13(5):101948. doi: 10.1016/j.gendis.2025.101948 (PMC13266120; doi:10.1016/j.gendis.2025.101948)
Supplement: Multimedia component 1 [file mmc1.docx]

**Figure S1** High expression levels of PI3K and HDAC-related genes are correlated with poor patient prognosis. **(A–E)** Kaplan–Meier analysis of progression-free survival was conducted using data from the Tumor Glioma French 284 database, with *P*-values obtained from the log-rank test indicated.

**Figure S2** Combination of CUDC-907 and temozolomide (TMZ) inhibits the migratory and invasive capabilities of glioblastoma cells. **(A)** Migration assay was performed after treatment with CUDC-907 and TMZ for 48 h. DMSO was used as a control. Scale bars = 100 μm. **(B)** Wound healing assay was performed with 24 h treatment of CUDC-907 and TMZ for 48 h. DMSO was used as a control. Scale bars = 1 mm. **(C)** The levels of N-Cadherin and Vimentin were assessed by Western blotting in LN229 and A172 cells following 48 h exposure to CUDC-907 and TMZ. DMSO was used as a control. The data were expressed as mean ± standard deviation. Student's *t*-test was performed to analyze significance. ^*^*P* < 0.05, ^**^*P* < 0.01, and ^***^*P* < 0.001.
